# Bristol PsychArts: A Psychiatry Film Club as a Wellbeing and Peer-Connection Intervention –A Two-Cycle QI Project Using Pre- and Post-Session Surveys

**DOI:** 10.1192/bjo.2026.11511

**Published:** 2026-06-30

**Authors:** Joanne Wagland, Jeremy Warner

**Affiliations:** Avon and Wiltshire Partnership NHS Trust, Bristol, United Kingdom

## Abstract

**Aims:**

Psychiatry trainees experience high workload and emotional demand. Peer-to-peer connection and the use of safe reflective spaces are protective factors, but access can be inconsistent. We used PDSA cycles to test whether an ongoing mental health-themed filmclub session (film, followed by facilitated discussion) was associated with immediate (same-evening) improvements in self-reported wellbeing and peer connection, and an immediate reduction in feelings of burnout.

**Methods:**

Setting: an open-to-all, mental health-themed film club with attendees including psychiatry trainees, other clinical staff, and non-clinicians. Intervention: film screening followed by a facilitated group discussion. Measures: brief anonymous surveys completed immediately pre- and post-session. Outcomes were: self-reported wellbeing, peer connection, and level of burnout. The post-session survey included an assessment of psychological safety during the session, plus free-text feedback for qualitative assessment. Likert responses in each domain were converted to numerical scores (1–5) and analysed quantitatively. After session 1, PDSA was used including reviewing qualitative feedback to inform refinements and confirm the post-film discussion as a key component.

**Results:**

Two cycles were completed with different films: Aftersun (2022) (session 1) and Taste of Cherry (1997) (session 2). Session 1: pre n=12, post n=11. Mean wellbeing increased from 3.17 to 4.00 (+0.83). Mean peer connection increased from 3.25 to 4.55 (+1.30). Mean burnout decreased from 2.92 to 2.73 (−0.19). Discussion comfort was high (mean 4.64/5). Session 2: pre n=13, post n=12. Mean wellbeing increased from 3.69 to 4.17 (+0.47). Mean peer connection increased from 4.38 to 4.75 (+0.37). Mean burnout decreased from 3.23 to 2.83 (−0.40). Psychological safety in the discussion remained high (mean 4.58/5; median 5). Free-text responses across both cycles repeatedly identified the post-film discussion as the main impact, describing peer-to-peer connection, widening of cultural perspectives, and a non-judgemental atmosphere.

**Conclusion:**

Across two QI cycles, a psychiatry film club was feasible to run and evaluate and was associated with immediate improvements in wellbeing and peer connection, with modest reductions in burnout feelings. These are key issues facing the mental health workforce and a film club represents a creative way to improve measures. Psychological safety ratings were consistently high and qualitative feedback suggests facilitated discussion as the key active component. Future cycles will look at sustained improvements in wellbeing. A future goal is to expand to other regions and embed into the core training curriculum.

